# Supplementary material for: Role of a single MCP in evolutionary adaptation of Shewanella putrefaciens for swimming in planktonic and structured environments
Source: Appl Environ Microbiol. 2025 Mar 25;91(4):e00229-25. doi: 10.1128/aem.00229-25 (PMC12016497; doi:10.1128/aem.00229-25)
Supplement: Supplemental material — Tables S1 to S3 and Figures S1 to S7. [file aem.00229-25-s0002.pdf]

Role of a single MCP in evolutionary adaptation of *Shewanella putrefaciens* for swimming in planktonic and structured environments

Daniel B Edelmann[1], Anna M Jakob[1], Laurence G Wilson[2], Remy Colin[3], David Brandt[4], Frederick Eck[1], Jörn Kalinowski[4], Kai M Thormann[1,*]

Affiliations:
[1]Institut für Mikrobiologie und Molekularbiologie, Justus-Liebig-Universität Gießen, Gießen, Germany
[2]Department of Physics, University of York, Heslington, York, YO10 5DD, UK.
[3]Max Planck Institute for Terrestrial Microbiology, and Center for Synthetic Microbiology (SYNMIKRO), Marburg, Germany
[4]Center for Biotechnology, Bielefeld University, Bielefeld, Germany

Supplementary Material

Supplementary Tables 1 – 3
Supplementary Figures 1 – 7
Caption to Supplementary Movie 1

Supplementary Table 1: Strains used in this study

Strain	Genotype	Source/Reference
Escherichia coli		
DH5α-λpir	φ80d*lacZ* ΔM15 Δ(*lacZYA-argF*)U169 *recA1 hsdR*17 *deoR thi*-l *supE*44 *gyrA*96 *relA*1/*λpir*	Miller and Mekalanos, 1988
WM3064	*thrB*1004 *pro thi rpsL hsdS lacZ*ΔM15 RP4-1360 Δ(*araBAD*) 567*ΔdapA* 1341::[erm pir(wt)]	W. Metcalf, University of Illinois at Urbana–Champaign, IL
Shewanella putrefaciens CN-32		
S271	CN-32 wild type	
	CN-32 Δ*flaAB*$_1$ Δ*flaAB*$_2$	(26)
	CN32 Δ*flaA*$_1$_T174C *B*$_1$_T166C_S174C Δf*laAB*$_2$	this study
	CN32 *flaA*$_1$_T174C *B*$_1$_T166C_S174C *flaA*$_2$_T159C *B*$_2$_T159C	this study
	14G clone 5 (evolved strain after 14 repeats)	this study
	14G clone 9 (evolved strain after 14 repeats)	this study
	14G clone 13 (evolved strain after 14 repeats)	this study
	14G clone 5 Δ24Δ0387	this study
	14G clone 9 Δ24Δ0387	this study
	14G clone 13 Δ24Δ0387	this study
	Δ24ΔCDS_0387	this study
	Δ24	this study
	CDS_0387-LuxCDABE	this study
	Δ24 CDS_0387-LuxCDABE	this study
	CDS_0387-mCherry	this study
	Δ24 CDS_0387-mCherry	this study

Supplementary Table 2: Plasmids used in this study

Name	Properties	Reference
pNPTS138-R6KT	*mobRP4*$^+$ *ori*-R6K *sacB*; suicide plasmid for in-frame deletions; Km^r	(86)
pNPTS138-R6KT_Δ*flaA*$_1$_T174C *B*$_1$_T166C_S174C	insert for flagellin gene modification in pNTPS138-R6K	this study
pNPTS138-R6KT_*flaA*$_2$_T159C *B*$_2$_T159C	insert for flagellin gene modification in pNTPS138-R6K	this study
pNPTS138-R6KT_Δ24ΔCDS_0387	insert for introduction of Δ24ΔCDS_0387 deletions in pNTPS138-R6K	this study
pNPTS138-R6KT_Δ24_CDS_0387knock-in	insert for re-insertion of Δ24ΔCDS_0387 in Δ24 in pNTPS138-R6K	this study
pNPTS138-R6KT_CDS_0387 mCherry	insert for introduction of a Sputcn32_0387-mCherry hybrid in pNTPS138-R6K	this study
pNPTS138-R6KT_CDS_0387 LuxCDABE	insert for introduction of a translational fusion of Sputcn32_0387-mCherry to *luxCDABE* in pNTPS138-R6K	this study
pBTOK	ori pBBR; oriT; Km^r; TetR, promoter and multiple cloning site of pASK-IBA3plus and *E. coli rrnB1* T1 and lambda phage T0	(28)

	terminator; overexpression plasmid for CN-32	
pBTOK_SP_0387	Sputcn32_0387 in pBTOK	this study

Supplementary Table 3: Oligonucleotides used in this study

Name	Construct/Purpose	Sequence
DE54	FlaA1_T174C_FlaB1_T166C_S174C	GAATTCGTGGATCCAGATtgaagttaaagtgtctggga
DE55	FlaA1_T174C_FlaB1_T166C_S174C	agttgcaatcgttaaACAactaaccattaaactccccg
DE56	FlaA1_T174C_FlaB1_T166C_S174C	agtttaatggttagtTGTttaacgattgcaacttcagg
DE57	FlaA1_T174C_FlaB1_T166C_S174C	aacttttaatgctgatgcACAggtttttgacacagaaatcgta
DE58	FlaA1_T174C_FlaB1_T166C_S174C	tcagcattaaaagttggtTGTttagatattaaaggctctgctcg
DE59	FlaA1_T174C_FlaB1_T166C_S174C	CAAGCTTCTCTGCAGGATctgtcacttcagataatttttcag
DE60	FlaA1_FlaB1_scr	tatctagacctgaccccatgcc
DE62	FlaA1_FlaB1_scr	aattttgatgcgactacccccg
DE01	FlaA2_C160_ FlaB2_C156	CAAGCTTCTCTGCAGGATGTCGCCGTCGCATTTTCG
DE24	FlaA2_C160_ FlaB2_C156	TTCCAAATTGGAGCTGGAaccGCAGAAtgtCTGGATGTGAAGTTAGGC
DE25	FlaA2_C160_ FlaB2_C156	ATCCAGACATTCTGCGgtTCCAGCTCCAATTTGGAA
DE26	FlaA2_C160_ FlaB2_C156	GCTGAAACATTGGCCGTTtgcacaACAGCTATCGATGACGCT
DE27	FlaA2_C160_ FlaB2_C156	ATCGATAGCTGTtgtgcaAACGGCCAATGTTTCAGC
DE10	FlaA2_C160_ FlaB2_C156	GCTGAAACATTGGCCGTTTGCTGTACAGCTATCGAT
VK184	FlaA2_FlaB2_scr	gttaccctttggcgcatcgg
VK183	FlaA2_FlaB2_scr	gtattagcttcgatcgggattgg
DE133	Δ24_Δ ORF0387	GAATTCGTGGATCCAGATccactgggcataaacctcaccc
DE134	Δ24_Δ ORF0387	ggataaagttttattatcggacagaaatgttaaaagttaaccttagtctgg
DE135	Δ24_Δ ORF0387	gataataaaactttatcccataaaaaacgccttagatggatgc
DE136	Δ24_Δ ORF0387	CAAGCTTCTCTGCAGGATagattggcaaatgcgtttatatgggcc
DE137	Δ24	ttgagatattttattatcggacagaaatgttaaaagttaaccttagtctgg
DE138	Δ24	ccgataataaaatatctcaaaacgtccaacacaggcatattga
DE139	CN32_0387_scr2	cctgccatttacgcctaaaggatagc
DE140	CN32_0387_scr1	cgccgcttagcctcgcag
DE198	0387_LuxCDABE	catatttgccctcctttataccttaaacttagccactaaagtatctaaacgatgg
DE199	0387_LuxCDABE	gctaagtttaaggtataaaggagggcaaatatgactaaaaaaatttcattcattattaacg
DE200	0387_LuxCDABE	ttttttatgggataaagtctAatcaaacgcttcggttaagctcaaagc
DE201	0387_LuxCDABE	accgaagcgtttgatTagactttatcccataaaaaacgccttagatggatg

DE192	0387Cterm-mCherry	GAATTCGTGGATCCAGATtagatgtgatccgcgctatctctgag
DE193	0387Cterm-mCherry	TTTGGAAACGCTCCCGCCtaccttaaacttagccactaaagtatctaaacgatgg
DE194	0387Cterm-mCherry	tttaaggtaGGCGGGAGCGTTTCCAAAGGGGAAGAGGACAATATGGC
DE195	0387Cterm-mCherry	tttatgggataaagtttaTTATTTGTATAACTCATCCATACCACCAGTCGAATG
DE196	0387Cterm-mCherry	GATGAGTTATACAAATAAtaaactttatcccataaaaaacgccttagatggatg
DE197	0387Cterm-scr	cgtgctcgcgataccattaaccaattg
FE128	pBTOK_SP_0387	AATTCGAGCTCGGTACCCgactaaggttaacttttaacatttct
FE129	pBTOK_SP_0387	CGACCTCGAGGGATCCCCttataccttaaacttagccactaaag

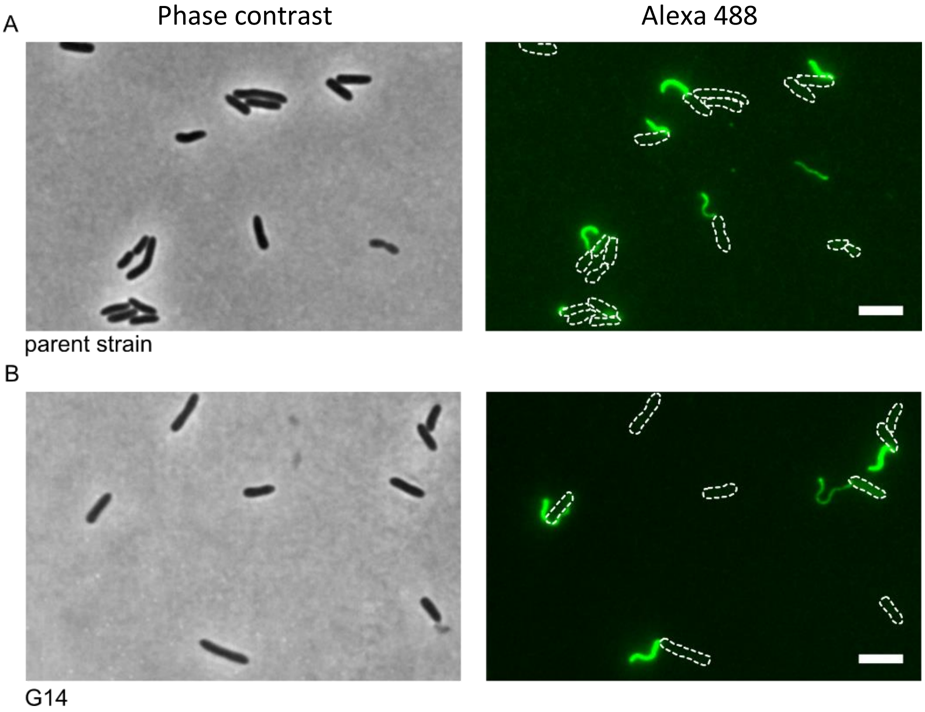

Supplementary Figure 1: Cell morphology and flagella staining of wild-type (A) and evolved (G14; B) mutant cells. Displayed are micrographs of cells with fluorescently labeled flagella, the cell positions are outlined in the fluorescent panels (right).

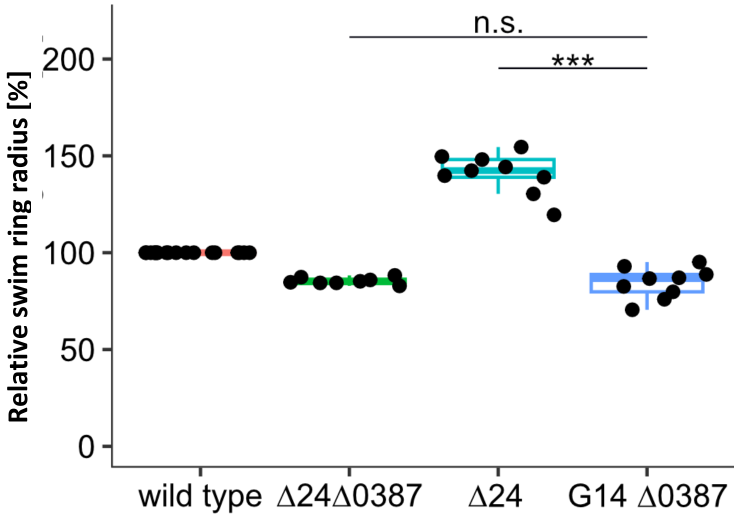

Supplementary Figure 2: Spreading phenotype of control strains as indicated. The asterisks indicate the significance according to a pairwise t-test ($p < 0.01$); n.s., not significant.

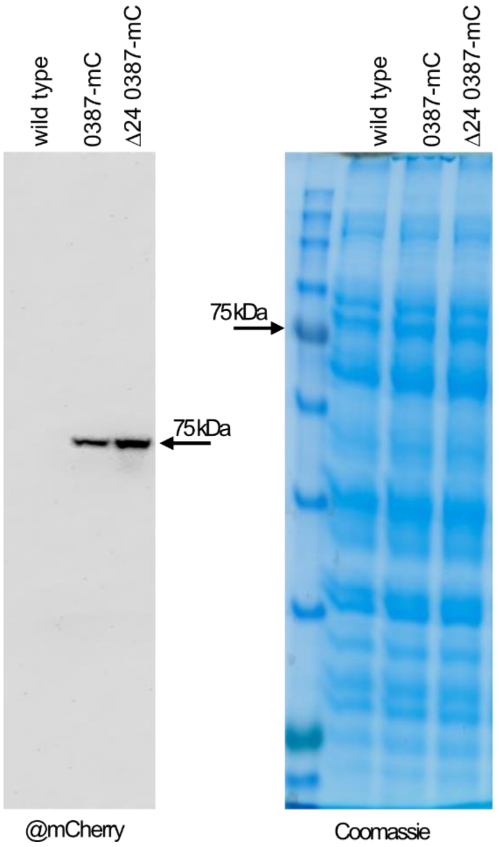

Supplementary Figure 3: Full western and loading control (Coomassie-stained PAGE) shown in main figure 3C. The mCherry fusion to MCP0387 results in a stable protein.

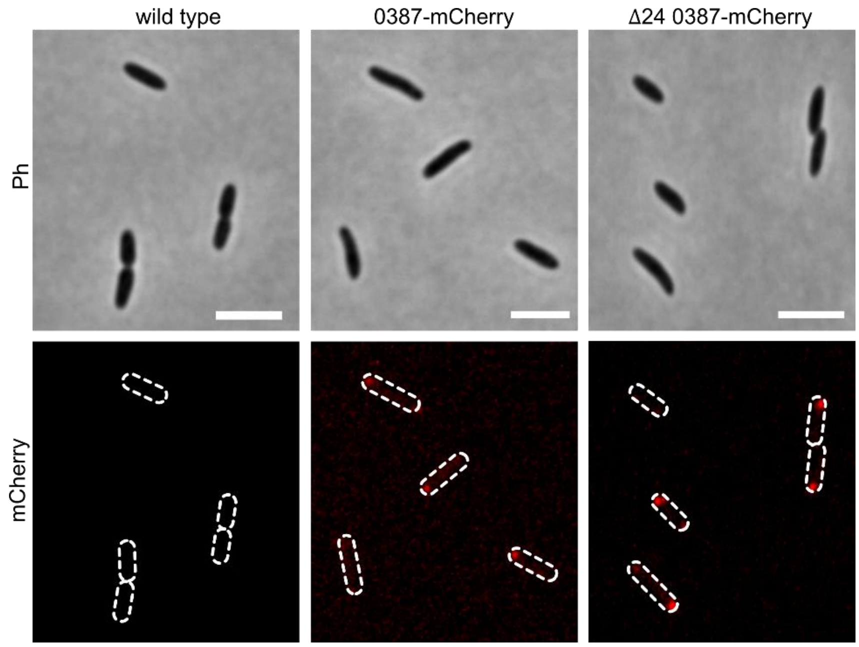

Supplementary Figure 4: Fluorescence microscopy including the wild type shown in main figure 3D. No signal occurs in non-tagged wild-type cells. The scale bars equal 5 µm.

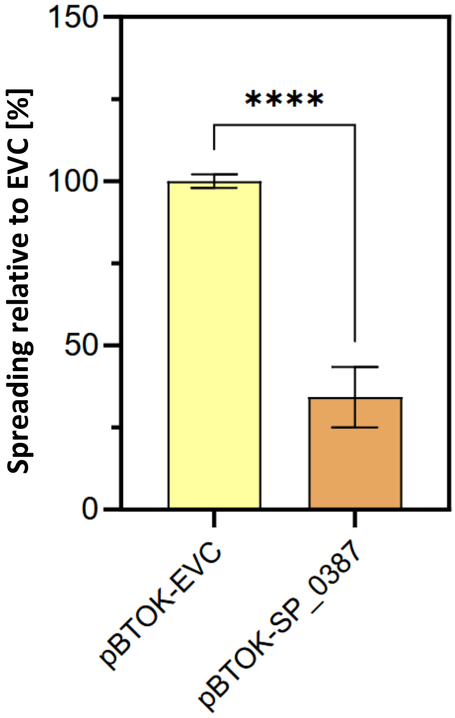

Supplementary Figure 5: Effect of MCP0387 on spreading through soft agar. pBTOK-EVC is the empty vector control, pBTOK-SP_0387 displays the overproduction. The asterisks indicate the significance according to a pairwise t-test ($p < 0.01$); n.s., not significant.

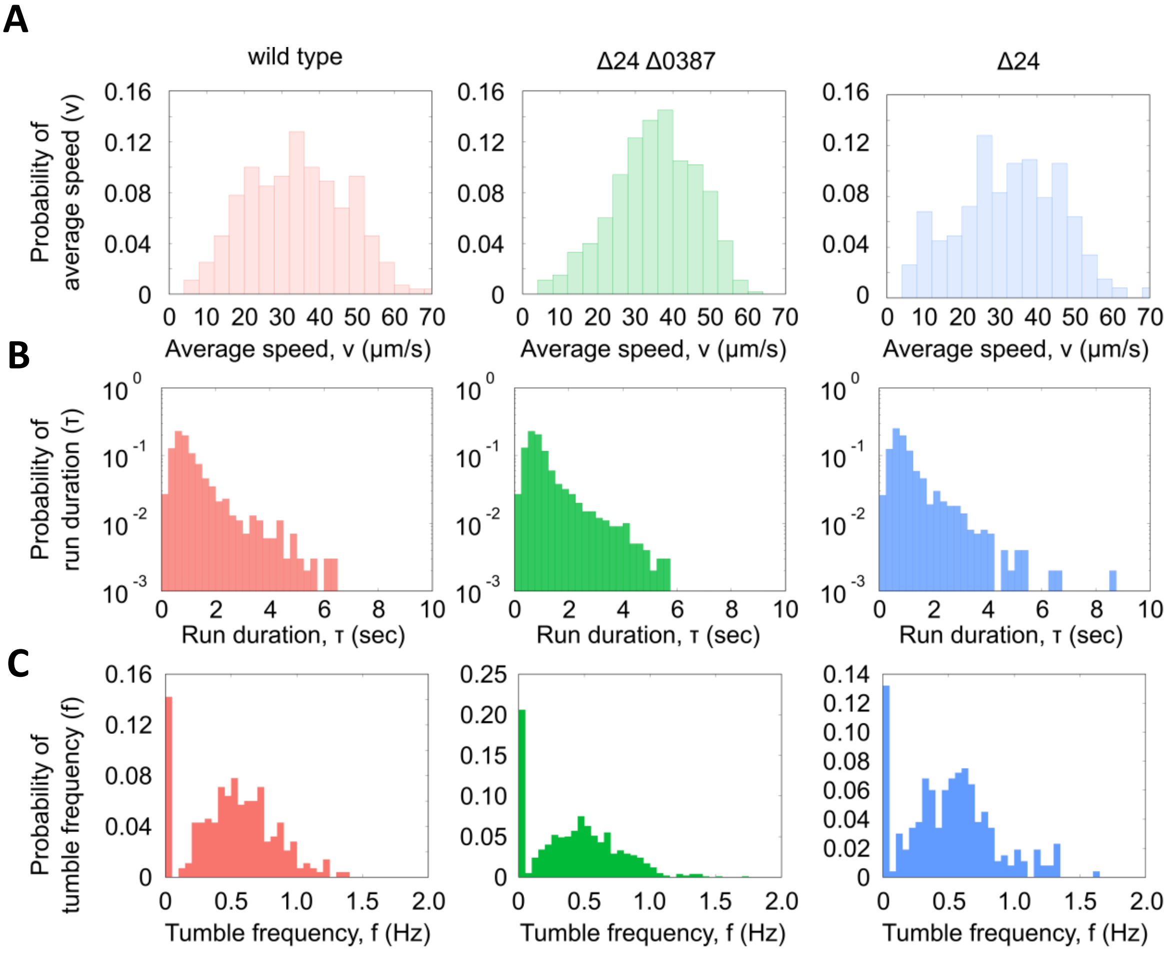

Supplementary Figure 6: Quantification of cell tracking under planktonic conditions. A) Distribution of track-averaged cell velocities (P(v)). The mean run speeds are 28±1 μm/s (WT, n=447), 31±1 μm/s (Δ24Δ0387, n=1233), and 26±1 μm/s (Δ24, n=365), where the uncertainties are 95% confidence intervals. **B)** Distribution of run lengths (P(τ)). **C)** Distribution of tumble frequencies (P(f)). The numbers of tracks analyzed were 281 for the wild type, 935 for the Δ24Δ0387 mutant and 265 for the Δ24 mutant.

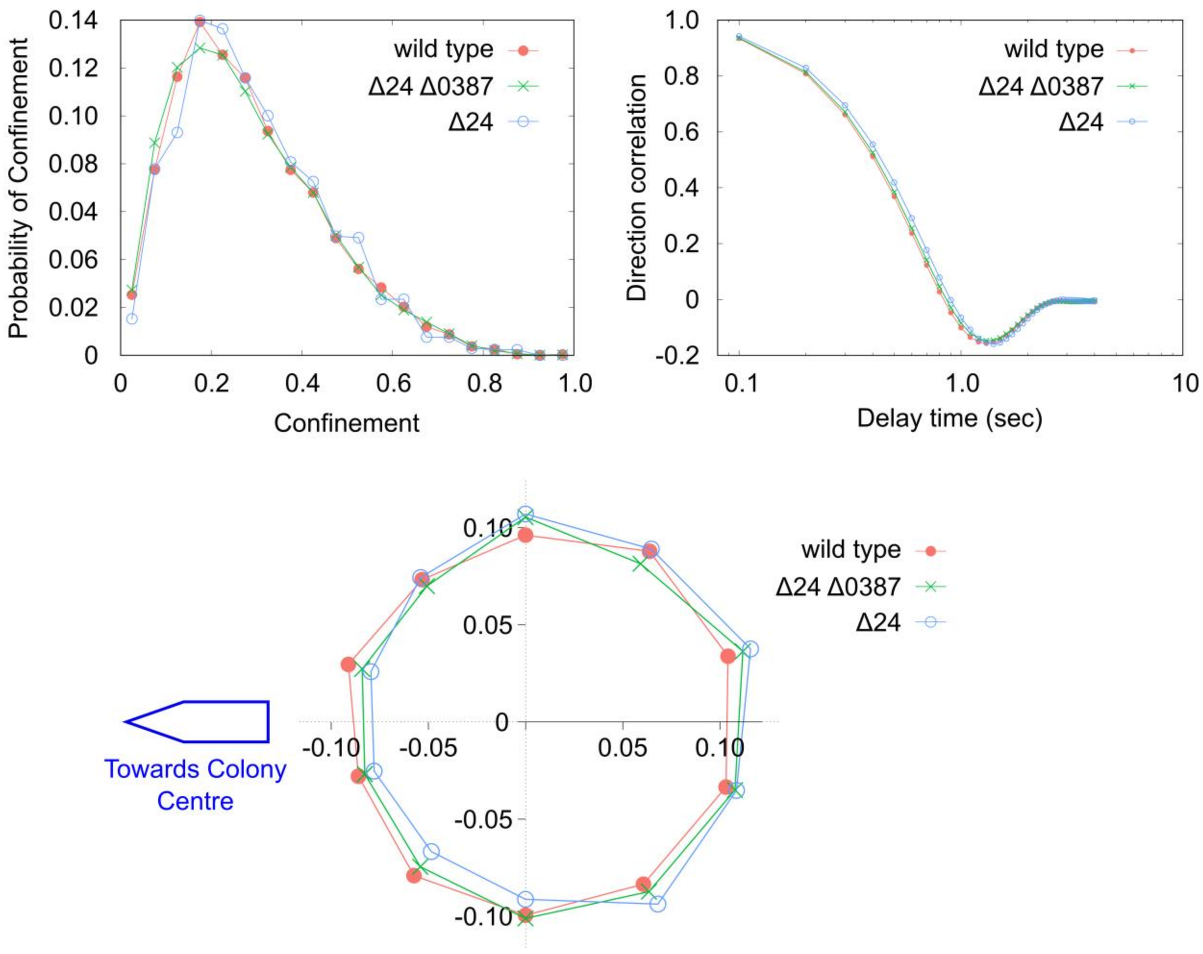

Supplementary Figure 7: Quantification of cell tracking in soft agar. A) Determination of the Confinement ratio, defined as ⟨**T**(t+τ) · **T**(t)⟩, where **T**(t) is the unit vector to the displacement between two subsequent frames. **B)** Determination of the angular distribution of steps for each strain, defined as a probability of motion in a particular direction. **C)** The net direction of each track was assessed by finding each track's end-to-end vector and allocating it to one of ten angular bins (width 36°). All cells show a roughly isotropic distribution of displacement directions, with a slight bias away from the colony center: the circles' centers are displaced slightly to the right of the origin. The data sets are normalized with respect to the number of tracks so that the sum of distances from the origin in each data set is 1, and the 95% confidence intervals are smaller than the points, as plotted. The number of tracks analyzed was 9,931 for the wild type, 11,393 for the Δ24Δ0387 mutant, and 1,709 for the Δ24 mutant.

Caption Supplementary Movie 1: Time-lapse scanning recording of soft-agar spreading assays using the indicated strains. Each petri dish was inoculated with a single culture and incubated at 30°C for 18h. The plates were scanned in 15-minutes time intervals.
